# Eligible Vehicles for Quinine

**Published:** 1890-10

**Authors:** 


### ELIGIBLE VEHICLES FOR QUININE.

Doubtless every pharmacist and physician has his favorite method of disguising the taste of unpalatable drugs, but not everyone is aware that the enterprise of manufacturing pharmacists now offers such a variety of vehicles from which to choose.

Some rely almost exclusively on pills and capsules, whereby the drug is smuggled into the stomach without recognition by the gustatory nerves. But there are patients whose apparatus for deglutition is so constructed as to render it almost impossible for them to swallow pills or capsules as well as cases where it is all important to secure immediate absorption of the medicine. When given in pill form, no matter how soluble the mass, an appreciable time must elapse before the remedy begins to have its effect.

How then can we administer quinine in solution or suspension—particularly to children or delicate ladies—without causing a disturbance in the family every time a dose has to be given? To children quinine may often be given advantageously by inunction, and the oleate of quinine especially, applied to the surface in this way, is readily absorbed, and produces promptly the characteristic effect of the drug. Suppositories must not be forgotton in cases where the stomach is particularly irritable, and the hypodermic injection presents itself as a dernier resort when a prompt and powerful influence is required. But in ordinary cases quinine may be administered by the mouth.

One plan is to mix the quinine with some alkali or astringent so that the bitter sulphate or muriate becomes converted into the tasteless alkaloid or tannate.

Another plan is to combine with the quinine a mixture having a bitterness of its own, which shall blend with and modify the intolerable bitterness of the quinine, some aromatic being generally added to still further disguise the objectionable taste.

It is on this principle that cascara cordial operates, and many of those who have tried this vehicle, declare that it is the best that has yet been offered. The especial advantage which it possesses over all others is the fact that it is a laxative agent, and so renders more efficient the action of the antiperiodic.

Licorice has long been known as having a remarbable power of covering the taste of bitter medicines. The property is due to a peculiar principle called glycyrrhizin, a glucoside, insoluble in water and in acid solutions, but readily dissolved by the aid of alkalies.

Where quinine is given in powders, it may be rendered nearly tasteless by simply rubbing it up with a small quantity (one-fourth its weight of ammoniated glycyrrhizin (ammoniated glycyrrhizate).

"Fluid extract licorice, for quinine mixtures," is one of the most efficient of all the preparations employed for covering the bitter taste of quinine. The best way to use it is to drop a dose of the powder into a little of the fluid extract contained in a spoon, mix it thoroughly and swallow at once.

Aromatic elixir of licorice is to be used in the same way as the fluid extract, but is especially useful in the drug store when a single dose of quinine is called for to be taken at once.

Yerba santa contains a principle analogous to glycyrrhizin, which renders quinine in its presence as tasteless as starch. It appears to act like glycyrrhizin by producing a peculiar impression upon the gustatory nerves; it does not, as stated by some, produce with the quinine an insoluble compound. Unless the mouth is thoroughly rinsed after taking the mixture, a bitter taste will gradually develop as the nerves recover from the influence of the yerba santa.

To some persons the taste of yerba santa itself is disagreeable, and when this is the case licorice is to be preferred. Barring, however, idiosyncrasy in this respect, we can recommend the preparations of yerba santa as the best means of rendering quinine tastless. Aromatic syrup of yerba santa renders it possible to give little children full doses of quinine without the vigorous remonstrances which physicians and parents have learned to regard as inevitable.—*Northwestern Pharmacist.*

